# Supplementary material for: The interactome of intact mitochondria by cross-linking mass spectrometry provides evidence for coexisting respiratory supercomplexes
Source: Mol Cell Proteomics. 2017 Dec 8;17(2):216–32. doi: 10.1074/mcp.RA117.000470 (PMC5795388; doi:10.1074/mcp.RA117.000470)
Supplement: Supplemental Data [file supp_RA117.000470_133922_0_supp_23978_fzffwf.zip › spectra_annotation/mito_DR_spectra_annotation/225-1-1-1-17-1.pdf]

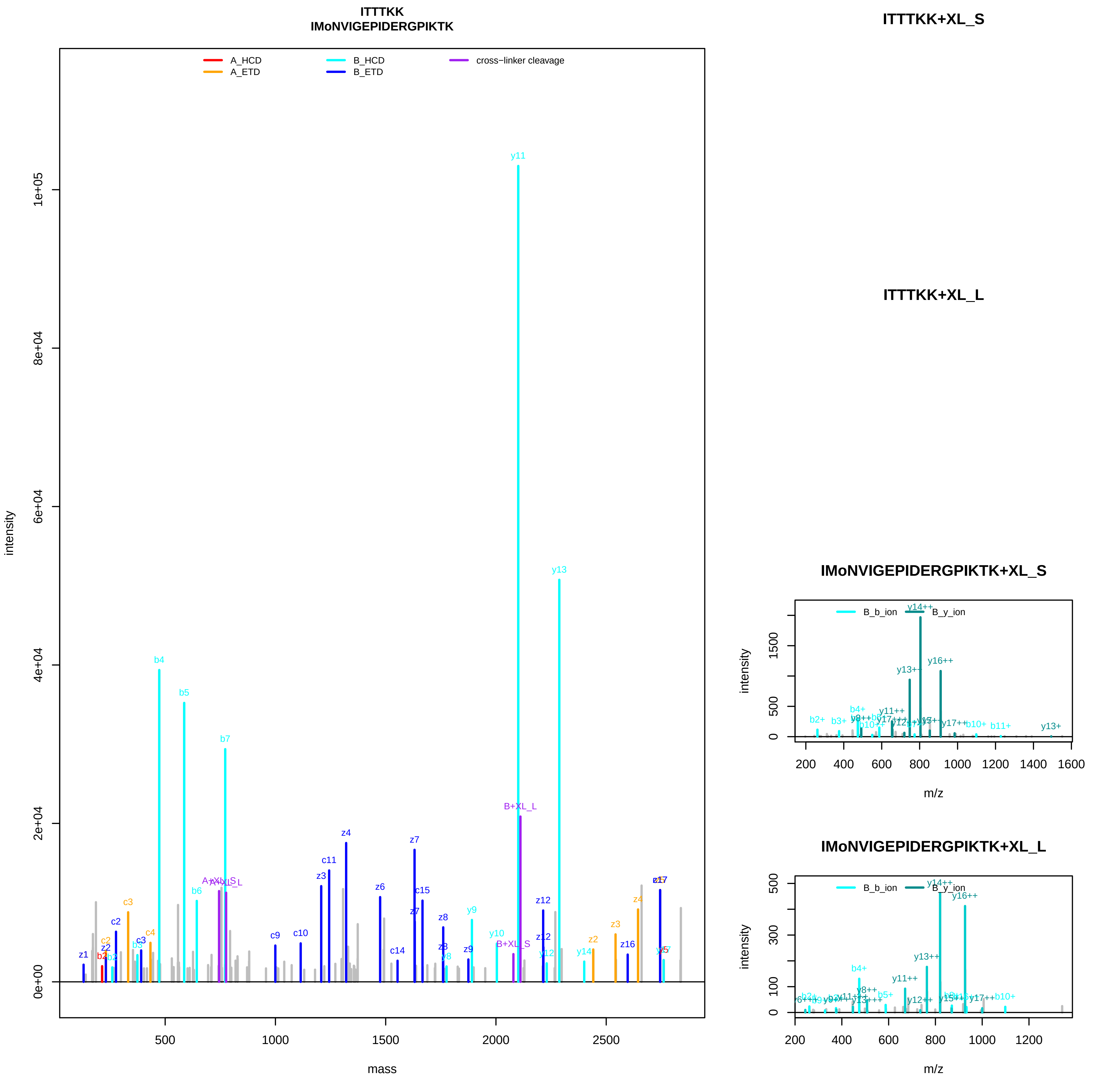
ITTTKK
IMoNVIGEPIDERGPIKTK
A_HCD
A_ETD
B_HCD
B_ETD
cross−linker cleavage
intensity
mass
0e+00
2e+04
4e+04
6e+04
8e+04
1e+05
500
1000
1500
2000
2500
y11
y13
b4
b5
b7
b6
B+XL_L
B+XL_S
A+XL_S
A+XL_L
z1
b2
c2
z2
c3
c4
b3
c9
c10
z3
c11
z4
z6
c14
z7
c15
z8
y8
z9
y9
y10
z12
y12
y14
z2
z3
z4
z16
z17
y5
y7
ITTTKK+XL_S
ITTTKK+XL_L
IMoNVIGEPIDERGPIKTK+XL_S
B_b_ion
B_y_ion
y14++
y16++
y13++
y11++
b4+
b2+
b3+
b10+
b11+
y13+
y17++
m/z
intensity
0
500
1500
200
400
600
800
1000
1200
1400
1600
IMoNVIGEPIDERGPIKTK+XL_L
B_b_ion
B_y_ion
y14++
y16++
y13++
b4+
y11++
y8++
b5+
y12++
y17++
b10+
0
100
300
500
200
400
600
800
1000
1200
m/z
intensity
